# Оценка распространенности анемий у пациентов с первичным гиперпаратиреозом: одноцентровое обсервационное исследование

**DOI:** 10.14341/probl12807

**Published:** 2021-09-30

**Authors:** А. П. Милютина, А. М. Горбачева, А. Р. Айнетдинова, А. К. Еремкина, Н. Г. Мокрышева

**Affiliations:** Национальный медицинский исследовательский центр эндокринологии; Российский национальный исследовательский медицинский университет им. Н.И. Пирогова; Национальный медицинский исследовательский центр эндокринологии; Национальный медицинский исследовательский центр эндокринологии; Национальный медицинский исследовательский центр эндокринологии; Национальный медицинский исследовательский центр эндокринологии

**Keywords:** анемия, первичный гиперпаратиреоз

## Abstract

ОБОСНОВАНИЕ. Сочетание первичного гиперпаратиреоза (ПГПТ) с анемией впервые было описано еще в 1931 г. Остается неясным, является ли ПГПТ непосредственной причиной возникновения анемии, или же она развивается вторично вследствие поражения других органов и систем. Частота анемий при ПГПТ в российской популяции неизвестна.ЦЕЛЬ. Оценить распространенность анемий у пациентов с ПГПТ, поступивших в отделение патологии околощитовидных желез ФГБУ «НМИЦ эндокринологии» Минздрава России в период с января 2017 по август 2020 г.МАТЕРИАЛЫ И МЕТОДЫ. В исследование вошли пациенты c ПГПТ старше 18 лет. Проведено одноцентровое обсервационное одномоментное одновыборочное неконтролируемое исследование. Анализировались лабораторно-инструментальные данные, полученные в ходе стационарного обследования пациентов с ПГПТ согласно стандартам оказания медицинской помощи больным с ПГПТ. Статистический анализ проведен в программных пакетах Statistica 13 (StatSoft, США) и SPSS (IBM, США).РЕЗУЛЬТАТЫ. В исследование включены 327 пациентов с ПГПТ, из них 28 (9%) мужчин и 299 (91%) женщин. Медиана возраста составила 59 лет [51; 66]. Среди всех обследованных пациентов по данным общеклинического анализа крови было выявлено 26 пациентов (8%) с анемией. В результате сравнения групп с анемией и без по различным параметрам биохимического анализа крови статистически значимые различия были обнаружены только по уровню скорости клубочковой фильтрации. В результате сравнения пациентов с анемией и без статистически значимых различий по частоте осложнений ПГПТ выявлено не было.Выявлены статистически значимые различия между пациентами с компрессионными переломами тел позвонков и без них по сывороточной концентрации гемоглобина и средней концентрации гемоглобина в эритроцитах. В ­группе пациентов без компрессионных переломов данные показатели были выше.В подгруппе пациентов с концентрацией общего кальция выше 3 ммоль/л и ПТГ выше 3 норм частота анемий достигала 21% (95% ДИ 10–35%). В данной подгруппе у пациентов с анемией выявлены тенденции к более высоким уровням ПТГ, ионизированного кальция и остеокальцина.ЗАКЛЮЧЕНИЕ. В целом взаимосвязи между гиперкальциемией, степенью повышения ПТГ и наличием анемии у пациентов с ПГПТ выявлено не было. Однако в подгруппе пациентов с выраженной гиперкальциемией (Са скорр. более 3,0 ммоль/л) отмечалась взаимосвязь между концентрациями паратиреоидного гормона, ионизированного кальция и наличием анемии. У пациентов с ПГПТ и компрессионными переломами тел позвонков наблюдались значимо более низкие концентрации гемоглобина крови и гемоглобина в эритроцитах.

## Abstract (en)

BACKGROUND: The combination of primary hyperparathyroidism (PHPT) with anemia was first described in 1931. It remains unclear whether PHPT is the direct cause of anemia, or it develops due to PHPT’s complications. The frequency of PHPT-­associated anemia in the Russian population is unknown.AIM: To assess the prevalence of anemia in patients with PHPT admitted to the Department of Parathyroid Glands Pathology in the Endocrinology Research Centre from January 2017 to August 2020.MATERIALS AND METHODS: The study included patients with PHPT over 18 years old. A single-center observational one-stage one-sample uncontrolled study was carried out. We analyzed laboratory and instrumental data obtained during inpatient examination in accordance with the standards of medical care. Statistical analysis was performed using Statistica 13 (StatSoft, USA) and SPSS (IBM, USA) software packages.RESULTS: The study included 327 patients with PHPT, 28 (9%) men and 299 (91%) women. The median age was 59 years [51; 66]. 26 patients (8%) with anemia were identified. Statistically significant differences between patients with and without anemia were found only in the GFR. Comparison of patients with and without anemia didn’t reveal any significant differences in the incidence of PHPT’s complications.Significant differences in serum hemoglobin concentration and average hemoglobin concentration in erythrocytes were revealed between patients with and without vertebrae fractures. In the group of patients without compression fractures these parameters were higher.In the subgroup of patients with total calcium concentration above 3 mmol/L and PTH above 3 normal values, the incidence of anemia reached 21% (95% CI: 10%; 35%). Within this group we revealed tendencies to higher levels of PTH, ionized calcium and osteocalcin in patients with anemia.CONCLUSION: In general, there was no correlation between hypercalcemia, the degree of PTH elevation and the presence of anemia in patients with PHPT. However, in the subgroup of patients with severe hypercalcemia, there was a relationship between the concentration of PTH, ionized calcium and the presence of anemia. In patients with PHPT and vertebral fractures, significantly lower concentrations of blood hemoglobin and hemoglobin in erythrocytes were observed.

## ОБОСНОВАНИЕ

Первичный гиперпаратиреоз (ПГПТ) — третья по распространенности эндокринопатия с заболеваемостью от 0,4 до 18,8 случаев на 10 000 населения в год. Для ПГПТ характерна избыточная секреция паратгормона (ПТГ) вследствие поражения околощитовидных желез (ОЩЖ), которая приводит к полиорганному поражению: остеопорозу, нефролитиазу, эрозиям, язвам желудочно-кишечного тракта и др. [1–5].

Сочетание ПГПТ с анемией впервые было описано D. Hunter [6] и J.C. Aub и соавт. [7]. Согласно этим и более поздним публикациям, частота анемий при ПГПТ колеблется в диапазоне 5–38%, при этом они чаще носят нормоцитарный и нормохромный характер [8–11]. Изменений в параметрах лейкопоэза и тромбоцитопоэза, как правило, не определяется [9][12].

Дискутабельным остается вопрос о том, является ли ПГПТ непосредственной причиной возникновения анемии, или же она развивается вторично вследствие поражения костной ткани, почек, эрозий или язв слизистой верхних отделов желудочно-кишечного тракта (ЖКТ). Частота анемий при ПГПТ в российской популяции неизвестна. Получение максимально полной информации о влиянии ПГПТ на развитие анемий позволило бы внести коррективы в тактику обследования и лечения этой когорты больных.

## ЦЕЛЬ ИССЛЕДОВАНИЯ

Целью исследования являлась оценка распространенности анемий у пациентов с ПГПТ, поступивших в отделение патологии ОЩЖ ФГБУ «НМИЦ эндокринологии» Минздрава России в период с января 2017 по август 2020 г.

## МАТЕРИАЛЫ И МЕТОДЫ

Место и время проведения исследования

Место проведения. Исследование было проведено на базе отделения патологии ОЩЖ ФГБУ «НМИЦ эндокринологии» Минздрава России.

Время исследования. С января 2017 по август 2020 гг.

Изучаемые популяции

В исследование вошли пациенты, находившиеся на стационарном лечении в отделении патологии ОЩЖ ФГБУ «НМИЦ эндокринологии» Минздрава России. Критериями включения было наличие у пациента подтвержденного диагноза ПГПТ, возраст старше 18 лет.

Критериями исключения являлись: гиперпаратиреоз любой другой этиологии, возраст младше 18 лет, отказ пациента от проведения стандартных клинико-лабораторных исследований по поводу ПГПТ.

Способ формирования выборки из изучаемой популяции

В данной работе применялся сплошной метод формирования выборки.

Дизайн исследования

Проведено одноцентровое обсервационное одномоментное неконтролируемое исследование. Анализировались лабораторно-инструментальные данные, полученные в ходе стационарного обследования пациентов с ПГПТ.

Методы

Анализировались параметры, включенные в стандарты оказания медицинской помощи больным с ПГПТ [13]:

- общеклинический анализ крови (оценивались концентрации гемоглобина, эритроцитов, лейкоцитов, тромбоцитов, гематокрит, средний объем эритроцита (MCV), среднее содержание гемоглобина в эритроците (MCH), средняя концентрация гемоглобина в эритроците (MCHC), ширина распределения эритроцитов (RDW), скорость оседания эритроцитов (СОЭ), лейкоциты, тромбоциты, 109 кл/л), исследования выполнены на анализаторе Sysmex XN-1000 (Sysmex Corp., Япония);

- биохимический анализ крови (кальций общий, ионизированный, альбумин-скорректированный, щелочная фосфатаза (ЩФ), остеокальцин, С-концевой телопептид коллагена I типа (СТХ), креатинин и скорость клубочковой фильтрации (СКФ)); исследованы на автоматическом биохимическом анализаторе ARCHITECT с8000 (Abbott, CША). Расчет альбумин-скорректированного кальция проводился по формуле:

общий кальций (ммоль/л) = измеренный уровень кальция сыворотки (ммоль/л) + 0,02 х (40 – измеренный уровень альбумина (г/л)).

СКФ определялась по формуле CKD-EPI 2009;

- паратгормон сыворотки крови (определение проводилось на электрохемилюминесцентном анализаторе Cobas 6000 (Roche, Германия));

- значения суточной кальциурии (автоматический биохимический анализатор ARCHITECT с8000 (Abbott, CША)).

Диагноз ПГПТ устанавливался или подтверждался согласно действующим клиническим рекомендациям [14]. Также согласно этому документу устанавливалось наличие костных и висцеральных осложнений основного заболевания.

Проводился скрининг костных и висцеральных осложнений ПГПТ [14]: рентгеновская денситометрия поясничного отдела позвоночника, бедренной и лучевой кости (Lunar iDXA, GE Healthcare, Япония или Hologic Discovery, США); рентгенография грудного и поясничного отделов позвоночника (рентгенодиагностическая система Optima RF420, GE Healthcare, Япония), ультразвуковое исследование (УЗИ) почек (аппарат Aplio 500, Toshiba, Япония) / мультиспиральная компьютерная томография (МСКТ) почек (компьютерный томограф GE Optima CT660, США); эзофагогастродуоденоскопия (ЭГДС) (видеодуоденоскоп Olympus GIF-XP 150N, Olympus Corporation, Япония).

Анемией считалось снижение концентрации гемоглобина ниже 120 г/л и/или числа эритроцитов менее 3,8×1012/л у женщин; ниже 130 г/л и/или числа эритроцитов менее 4×1012 /л — у мужчин [15].

Статистический анализ

Статистический анализ проведен в программных пакетах Statistica 13 (StatSoft, США) и SPSS (IBM, США).

Для определения соответствия распределения количественных данных нормальному закону использовался тест Шапиро–Уилка. Описательная статистика количественных показателей представлена медианами, первым и третьим квартилями в виде Me [Q1; Q3]. Описательная статистика качественных показателей представлена в виде абсолютных и относительных частот.

Сравнение двух независимых групп для количественных данных выполнялось с помощью критерия Манна–Уитни (U-тест). Частоты бинарных признаков сравнивались между собой с помощью критерия Хи-квадрат (χ2). При необходимости применялась поправка Йейтса.

Критический уровень статистической значимости при проверке статистических гипотез принят равным 0,05. При множественных сравнениях применялась поправка Бонферрони путем коррекции критического уровня значимости. Рассчитанные уровни значимости в диапазоне от критического до 0,05 описаны в качестве индикаторов статистической тенденции.

ДИ для частот рассчитывались методом Клоппера–Пирсона.

Этическая экспертиза

Протокол исследования одобрен локальным этическим комитетом ФГБУ «НМИЦ эндокринологии» Минздрава России. Было постановлено одобрить возможность проведения одноцентрового обсервационного одномоментного одновыборочного неконтролируемого исследования, посвященного оценке распространенности анемий у пациентов с ПГПТ, поступивших в отделение патологии околощитовидных желез ФГБУ «НМИЦ эндокринологии» Минздрава России в период с января 2017 по август 2020 г. Научная работа соответствует этическим стандартам добросовестной клинической практики (протокол № 16 от 11.08.2021).

## РЕЗУЛЬТАТЫ

Описание выборки

В исследование включены 327 пациентов с ПГПТ, из них 28 (9%) мужчин и 299 (91%) женщин. Медиана возраста составила 59 лет [ 51; 66 ]. Характеристика пациентов на момент госпитализации представлена в таблице 1.

Частота анемий в исследуемой выборке

Среди всех обследованных пациентов по данным общеклинического анализа крови было выявлено 26 пациентов (8%; 95% ДИ 5–11%) с анемией. Медиана возраста в группе пациентов с анемией составила 64 года [ 50; 72 ], в группе без анемий — 59 лет [ 51; 66 ]. Группы были сопоставимы по полу и возрасту (р=0,097; χ2 с поправкой Йейтса; р=0,131; U-тест).

Связь частоты анемии с биохимическими параметрами крови

В результате сравнения групп с анемией и без по различным параметрам биохимического анализа крови с учетом поправки Бонферрони (Р0=0,005) статистически значимые различия были обнаружены только по уровню СКФ, также были выявлены тенденции к различиям по уровню суточной кальциурии и сывороточной концентрации креатинина (табл. 2).

Связь частоты анемии с наличием осложнений ПГПТ

В результате сравнения пациентов с анемией и без статистически значимых различий по частоте осложнений ПГПТ выявлено не было (табл. 3).

Далее была выделена группа пациентов с уровнем общего кальция выше 3 ммоль/л и ПТГ выше 3 норм как наиболее тяжелая. Частота анемий среди таких пациентов — 10/48 (21%; 95% ДИ 10–35%). В результате сравнения подгрупп с анемией и без внутри данной группы по параметрам фосфорно-кальциевого обмена с учетом поправки Бонферрони (Р0=0,006) были выявлены тенденции к различиям по уровню ПТГ, ионизированного кальция и остеокальцина (табл. 4).

Частота анемий у пациентов с костными осложнениями ПГПТ

Костные осложнения ПГПТ были выявлены у 204 пациентов (63%; 95% ДИ 57–68%). Анемия в данной когорте была диагностирована у 17 больных (8,33%), в группе пациентов без костных осложнений — у 9 (7,32%). Данные группы сравнивались по параметрам общеклинического анализа крови (табл. 5).

С учетом поправки Бонферрони (Р0=0,005) выявлены статистические тенденции к различиям по количеству эритроцитов и среднему объему эритроцитов в крови: в группе пациентов с костными осложнениями наблюдалось снижение их числа и увеличение объема.

Частота анемий у пациентов с компрессионными переломами тел позвонков

У 51 пациента (16%; 95% ДИ 12–20%) были выявлены компрессионные переломы тел позвонков. Анемия в данной группе была диагностирована у 7 пациентов (13,73%), в группе пациентов без костных осложнений — у 19 (6,88%). Данные группы были также сравнены по результатам общеклинического анализа крови (табл. 6).

С учетом поправки Бонферрони (Р0=0,005) выявлены статистически значимые различия по сывороточной концентрации гемоглобина и средней концентрации гемоглобина в эритроцитах. В группе пациентов без компрессионных переломов данные показатели были выше. Также были обнаружены статистические тенденции к снижению гематокрита, количества эритроцитов, распределению эритроцитов по объему в группе с костными осложнениями. СОЭ в данной группе пациентов имела тенденцию к повышению.

**Table:** Таблица 1. Характеристика пациентов с первичным гиперпаратиреозом на момент госпитализации

Показатель	Число пациентов	Me [Q1; Q3]
Общий клинический анализ крови
Гемоглобин, г/л	327	138 [ 129; 147]
Эритроциты, 1012 кл./л	327	4,71 [ 4,42; 4,97]
Гематокрит, %	327	42,5 [ 39,6; 44,6]
MCV, фл	327	90,0 [ 87,5; 92,7]
MCH, пг	327	29,5 [ 28,4; 30,5]
MCHC, г/л	327	327 [ 319; 333]
RDW, %	327	13,1 [ 12,7; 13,9]
СОЭ, мм/ч	327	10 [ 5; 18]
Лейкоциты, 109 кл/л	327	6,04 [ 5,06; 6,96]
Тромбоциты, 109 кл/л	327	242 [ 208; 280]
Биохимический анализ крови
Кальций общий, ммоль/л	326	2,75 [ 2,65; 2,94]
Кальций ионизированный, ммоль/л	316	1,30 [ 1,24; 1,37]
Альбумин-скорректированный кальций, ммоль/л	326	2,67 [ 2,57; 2,86]
ПТГ, пг/мл	324	132,4 [ 101,7; 219,0]
Суточная кальциурия, ммоль/сут	305	7,70 [ 5,16; 10,41]
ЩФ, Ед/л	286	88,0 [ 70,0; 115,5]
Остеокальцин, нг/мл	268	40,96 [ 25,00; 67,98]
СТХ, нг/мл	267	0,74 [ 0,44; 1,23]
Креатинин, мкмоль/л	325	68,3 [ 62,2; 75,0]
СКФ, мл/мин/1,73 м2	325	84 [ 76; 95]
Осложнения ПГПТ
Компрессионные переломы	327	51 (16,6%)
Внепозвоночные остеопоротические переломы	327	111 (33,94%)
Костные осложнения	327	204 (62,39%)
Нефрокальциноз/нефролитиаз	327	165 (50,46%)
Эрозивно-язвенное поражение ЖКТ	296	61 (20,61%)

**Table:** Таблица 2. Сравнение групп пациентов с/без анемии с первичным гиперпаратиреозом по биохимическому анализу крови

Показатель	Анемии есть	Анемий нет	р, U-тест
n	Me [Q1; Q3]	n	Me [Q1; Q3]
Кальций общий, ммоль/л	26	2,74 [ 2,55; 3,09]	300	2,75 [ 2,65; 2,92]	0,788
Кальций ионизированный, ммоль/л	24	1,32 [ 1,22; 1,51]	292	1,30 [ 1,24; 1,36]	0,389
Альбумин-скорректированный кальций, ммоль/л	26	2,71 [ 2,56; 3,06]	301	2,670 [ 2,57; 2,83]	0,481
ПТГ, пг/мл	26	253,0 [ 101,6; 497,3]	298	131,1 [ 101,8; 208,7]	0,062
Суточная кальциурия, ммоль/сут.	18	5,412 [ 2,457; 7,790]	287	7,76 [ 5,28; 10,49]	0,018
ЩФ, Ед/л	22	102,5 [ 76,8; 178,0]	264	88,0 [ 69,0; 112,2]	0,098
Остеокальцин, нг/мл	23	67,85 [ 26,27; 141,45]	245	40,64 [ 24,77; 59,47]	0,071
СТХ, нг/мл	23	1,02 [ 0,63; 1,51]	244	0,73 [ 0,41; 1,21]	0,115
Креатинин, мкмоль/л	26	78,1 [ 63,9; 165,9]	299	68,0 [ 62,2; 73,5]	0,009
СКФ, мл/мин/1,73 м2	26	68 [ 32; 91]	299	84 [ 77; 96]	0,002

**Table:** Таблица 3. Сравнение групп пациентов с/без анемии и по частоте развития осложнений первичного гиперпаратиреоза

Показатель	Анемии есть	Анемий нет	р, χ2
абс. частота (%) n=26	абс. частота (%) n=301
Компрессионные переломы	7 (26,92)	44 (14,62)	0,168*
Внепозвоночные остеопоротические переломы	11 (42,31)	100 (33,22)	0,348
Костные осложнения	17 (65,38)	187 (62,13)	0,607
Нефрокальциноз/нефролитиаз	15 (57,69)	150 (49,83)	0,442

**Table:** Таблица 4. Сравнение подгрупп пациентов с/без анемии с тяжелым течением первичного гиперпаратиреоза по параметрам фосфорно-кальциевого обмена

Показатель	Анемии есть	Анемий нет	р, U-тест
n	Me [Q1; Q3]	n	Me [Q1; Q3]
ПТГ, пг/мл	10	571,95[ 423,60; 1027,00]	38	383,30[ 265,80; 567,10]	0,024
Кальций общий, ммоль/л	10	3,32[ 3,17; 3,55]	38	3,26[ 3,08; 3,35]	0,203
Кальций ионизированный, ммоль/л	10	1,54[ 1,52; 1,65]	35	1,49[ 1,43; 1,57]	0,038
Альбумин-скорректированный кальций, ммоль/л	10	3,28[ 3,08; 3,43]	38	3,16[ 3,00; 3,26]	0,065
Суточная кальциурия, ммоль/сут	7	7,95[ 3,40; 8,88]	36	9,90[ 7,99; 12,94]	0,097
Фосфор	10	0,86[ 0,66; 0,95]	38	0,75[ 0,63; 0,81]	0,082
ЩФ, Ед/л	10	152,50[ 106,00; 266,00]	33	128,00[ 105,00; 244,00]	0,788
Остеокальцин, нг/мл	8	213,05[ 124,60; 300,00]	31	106,50[ 52,98; 159,70]	0,023
СТХ, нг/мл	8	1,755[ 1,32; 3,56]	31	1,53[ 1,18; 2,16]	0,441

**Table:** Таблица 5. Сравнение групп пациентов с костными осложнениями и без с первичным гиперпаратиреозом по параметрам общеклинического анализа крови

Показатель	Костные осложнения есть	Костных осложнений нет	р, U-тест
n	Me [Q1; Q3]	n	Me [Q1; Q3]
Гемоглобин, г/л	204	138,0 [ 129,0; 145,0]	123	140,0 [ 129,0; 149,0]	0,094
Эритроциты, 1012 кл/л	204	4,68 [ 4,39; 4,92]	123	4,78 [ 4,47; 5,06]	0,013
Гематокрит, %	204	42,20 [ 39,60; 44,40]	123	43,00 [ 39,60; 44,90]	0,144
MCV, фл	204	90,80 [ 88,10; 93,12]	123	89,00 [ 86,85; 91,85]	0,006
MCH, пг	204	29,60 [ 28,57; 30,60]	123	29,20 [28,20; 30,40]	0,067
MCHC, г/л	204	326,0 [ 318,8; 332,0]	123	327,0 [ 321,0; 333,5]	0,205
RDW, %	204	13,10 [ 12,70; 13,62]	123	13,10 [ 12,60; 14,20]	0,825
СОЭ, мм/ч	204	11,00 [ 5,75; 19,25]	123	10,00 [ 5,00; 16,50]	0,205
Лейкоциты, 109 кл/л	204	5,93 [ 4,95; 6,87]	123	6,280 [ 5,160; 7,505]	0,051
Тромбоциты, 109 кл/л	204	237,5 [ 206,0; 280,0]	123	248,0 [ 214,0; 281,0]	0,150

**Table:** Таблица 6. Сравнение групп пациентов с компрессионными переломами и без с первичным гиперпаратиреозом по параметрам общеклинического анализа крови

Показатель	Компрессионные переломы есть	Компрессионных переломов нет	р, U-тест
n	Me [Q1; Q3]	n	Me [Q1; Q3]
Гемоглобин, г/л	51	132,0 [ 125,0; 142,5]	276	139,5 [ 130,0; 148,0]	<0,001
Эритроциты, 1012 кл/л	51	4,59 [ 4,40; 4,87]	276	4,74 [ 4,43; 4,99]	0,039
Гематокрит, %	51	41,30 [ 38,85; 43,55]	276	42,70 [ 39,67; 44,70]	0,025
MCV, фл	51	90,40 [ 88,00; 93,35]	276	90,00 [ 87,38; 92,70]	0,554
MCH, пг	51	29,00 [ 28,25; 30,15]	276	29,50 [ 28,40; 30,62]	0,111
MCHC, г/л	51	321,0 [ 316,0; 326,0]	276	328,0 [ 320,0; 333,2]	<0,001
RDW, %	51	13,40 [ 13,05; 13,90]	276	13,00 [ 12,70; 13,90]	0,006
СОЭ, мм/ч	51	13,00 [ 7,00; 22,00]	276	10,00 [ 5,00; 18,00]	0,015
Лейкоциты, 109 кл/л	51	5,930 [ 5,220; 6,685]	276	6,08 [ 5,04; 7,09]	0,564
Тромбоциты, 109 кл/л	51	251,0 [ 199,5; 282,5]	276	241,0 [ 208,0; 279,0]	0,862

## ОБСУЖДЕНИЕ

Репрезентативность выборок

Набор участников проводился только в ФГБУ «НМИЦ эндокринологии» Минздрава России, что ограничивает репрезентативность полученной выборки.

Сопоставление с другими публикациями

Исследования, посвященные изучению анемий у пациентов с ПГПТ, крайне лимитированы. Так, S.K. Bhadada и соавт. [16] выявили анемию у 15 из 28 (53,3%) пациентов с манифестным течением ПГПТ, а M. Boxer и соавт. [9] — у 17 из 332 (5,1%) больных. В исследовании J.M. Falko и соавт. [12] анемия была диагностирована у 5 из 100 больных, и только у 2 из них она не была обусловлена другими причинами, такими как алиментарный дефицит железа или фолатов.

Наши данные в наибольшей степени соответствуют данным M. Boxer (5,1%) — среди наших больных с ПГПТ анемия была выявлена у 8%.

Однако в наш анализ были включены пациенты как с манифестным заболеванием, так и с мягким и/или нормокальциемическим течением ПГПТ. Ионы кальция являются важным регулятором эритропоэза: ПТГ увеличивает поступление ионов Ca2+ внутрь эритроидных предшественников, снижая их пролиферацию, и внутрь самих эритроцитов, снижая их осмотическую устойчивость [17][18]. На основании данных S.K. Bhadada и соавт. [16] мы предположили, что частота анемий при ПГПТ может коррелировать с тяжестью основного заболевания. В работе M. Boxer и соавт. [9] у пациентов с анемией определялись значимо более высокие сывороточные концентрации ПТГ, ЩФ и ионизированного кальция. В нашем исследовании корреляций между степенью выраженности гиперкальциемии и наличием анемии в общей выборке выявлено не было; среди лабораторных исследований взаимосвязь с наличием анемии была получена только для СКФ. Это согласуется с классическими представлениями о регуляции гемопоэза, а именно с выработкой эритропоэтина почками и снижением этой продукции по мере угнетения СКФ [19]. Отсутствие корреляции между выраженностью гиперкальциемии и наличием анемии, вероятно, может быть обусловлено тем, что на момент поступления в стационар пациенты с клинически наиболее тяжелым течением заболевания уже получали медикаментозную терапию. Это подтверждается относительно невысокими медианами кальциемии в обеих группах: 2,67–2,71 ммоль/л (см. табл. 2).

В то же время при анализе подгруппы пациентов с выраженной гиперкальциемией (Са скорр. более 3,0 ммоль/л, см. табл. 4) анемия чаще выявлялась у пациентов с более высокими сывороточными концентрациями ПТГ.

Висцеральные осложнения ПГПТ (такие как нефролитиаз со снижением фильтрационной функции почек, эрозии/язвы верхних отделов ЖКТ) могут являться этиопатогенетическими факторами развития анемии; при этом при нормализации показателей минерального обмена достигается и ремиссия анемии [12][20][21].

Однако имеются данные о развитии анемии у пациентов с ПГПТ с сохранной СКФ и без признаков острой или хронической кровопотери [22]. Ведущим механизмом таких изменений может являться миелофиброз [23–25].

В работе S.K. Bhadada и соавт. [16] у 75% больных с анемией при ПГПТ, которым была выполнена трепанобиопсия костного мозга, был диагностирован миелофиброз. Интересно, что после паратиреоидэктомии достигалось не только повышение концентрации гемоглобина, но и уменьшение выраженности миелофиброза. Взаимосвязи между выраженностью миелофиброза и длительностью течения ПГПТ, повышением сывороточных концентраций кальция и ПТГ авторами выявлено не было. В уже упоминавшейся работе M. Boxer и соавт. [9] трепанобиопсия была выполнена 5 из 17 пациентам с анемией при ПГПТ, у 4 больных был диагностирован миелофиброз различной степени выраженности, а у одного — его начальные проявления. В то же время рутинно трепанобиопсия пациентам с ПГПТ не проводится, что не позволяет делать выводы об истинной распространенности миелофиброза в данной когорте больных.

Косвенно о вовлеченности костномозгового кроветворения в патологический каскад при ПГПТ можно судить по состоянию костной системы в целом. В нашем исследовании у пациентов с костными осложнениями ПГПТ наблюдалась тенденция к снижению числа и увеличению объема эритроцитов. При тяжелом остеопорозе — наличии компрессионных переломов тел позвонков — статистически значимо ниже были сывороточная концентрация гемоглобина и средняя концентрация гемоглобина в эритроцитах. Существует ряд работ, подтверждающих, что анемия является независимым фактором риска переломов в целом и внепозвоночных переломов в частности [26][27], но не переломов тел позвонков [28]. Таким образом, на данном этапе однозначно интерпретировать полученные результаты не представляется возможным.

Влияние избыточной секреции ПТГ на эритропоэз, по данным литературы, также реализуется за счет ингибирования эритроидных клеток-предшественниц и прямого подавляющего эффекта на синтез эритропоэтина [17][18]. При этом механизмы развития анемии при первичном и вторичном гиперпаратиреозе существенно разнятся, поскольку при ПГПТ и сохранной СКФ отсутствует эндогенный дефицит эритропоэтина [17].

Избыточные концентрации ПТГ могут приводить к тромбоцитопатии (снижению агрегации тромбоцитов), ассоциированной с повышенной кровопотерей и, как следствие, анемией [25][29]. Имеются данные о том, что средний объем тромбоцитов (MPV) снижается у пациентов с ПГПТ после паратиреоидэктомии, а сам MPV прямо коррелирует с концентрацией ПТГ и кальция в сыворотке крови [30]. В нашей работе отличий по содержанию тромбоцитов в крови пациентов выявлено не было, MPV не оценивался.

Влияния ПГПТ на лейкопоэз ранее выявлено не было [9][16], что согласуется с нашими данными.

Клиническая значимость результатов

Полученные в нашем исследовании данные позволили впервые оценить распространенность анемии среди пациентов с ПГПТ, госпитализированных в специализированный стационар. На основании проведенной работы будут спланированы дальнейшие исследования, которые позволят установить показания к активному скринингу нарушений гемопоэза при ПГПТ.

Ограничения исследования

Проведенное исследование является пилотным, носит описательный характер и имеет ряд ограничений.

Выборка пациентов была сформирована на базе одного центра, в связи с чем не отражает истинный портрет пациента в российской популяции: чаще всего в учреждения третьего уровня направляются пациенты с тяжелым/нетипичным течением заболевания. Полученные данные позволяют пилотно оценить распространенность анемии среди пациентов с ПГПТ, поступивших в стационар.

В рамках госпитализации было недоступно проведение расширенного скрининга состояния эритропоэза: анализы крови на железо, витамины В6 и В12, трансферрин, общая железосвязывающая способность сыворотки, эритропоэтин и др. Это ограничивает нас в интерпретации причин анемии и установления ее патогенетической связи с ПГПТ.

Также необходима более детализированная оценка состояния костной ткани этих больных: включение в статическую обработку параметров BMD позволит точнее установить степень поражения костной ткани.

Оценка наличия миелофиброза также не проводилась в связи с отсутствием как технической возможности, так и (чаще всего) показаний к данной инвазивной процедуре.

Направления дальнейших исследований

В дальнейшем планируется проведение проспективного исследования состояния эритропоэза у пациентов с ПГПТ до и после хирургического лечения с расширением списка анализируемых показателей. Кроме того, значимый вклад в понимание влияния ПГПТ на гемопоэз могут внести фундаментальные работы по оценке течения эритропоэза в культуре клеток/тканей в условиях гиперкальциемии и повышенной концентрации ПТГ.

## ЗАКЛЮЧЕНИЕ

Взаимосвязи между гиперкальциемией, степенью повышения ПТГ и наличием анемии у пациентов с ПГПТ выявлено не было. Однако в подгруппе пациентов с выраженной гиперкальциемией (Са скорр. более 3,0 ммоль/л) отмечалась взаимосвязь между концентрацией ПТГ, ионизированного кальция и наличием анемии. У пациентов с ПГПТ и компрессионными переломами тел позвонков наблюдались значимо более низкие концентрации гемоглобина крови и гемоглобина в эритроцитах.

## ДОПОЛНИТЕЛЬНАЯ ИНФОРМАЦИЯ

Источники финансирования. Публикация настоящей работы поддержана государственным заданием. Номер государственного учета НИОКТР АААА-А20-121030100032-7.

Конфликт интересов. Авторы декларируют отсутствие явных и потенциальных конфликтов интересов, связанных с содержанием настоящей статьи

Участие авторов. Милютина А.П. — сбор данных, статистический расчет и интерпретация результатов, написание текста рукописи; Горбачева А.М. — концепция и дизайн исследования, сбор данных и интерпретация результатов, написание текста рукописи; Айнетдинова  А.Р. — сбор данных, статистический расчет и интерпретация результатов, написание текста рукописи; Еремкина А.К. — концепция и дизайн исследования, интерпретация результатов, написание текста рукописи; Мокрышева Н.Г. — написание текста рукописи. Все авторы одобрили финальную версию статьи перед публикацией, выразили согласие нести ответственность за все аспекты работы, подразумевающую надлежащее изучение и решение вопросов, связанных с точностью или добросовестностью любой части работы.

## References

[cit1] Yeh Michael W., Ituarte Philip H. G., Zhou Hui Cynthia, Nishimoto Stacie, Amy Liu In-Lu, Harari Avital, Haigh Philip I., Adams Annette L. (2013). Incidence and Prevalence of Primary Hyperparathyroidism in a Racially Mixed Population. The Journal of Clinical Endocrinology & Metabolism.

[cit2] Adami S, Marcocci C, Gatti D. Epidemiology of primary hyperparathyroidism in Europe. J Bone Miner Res. 2002;17 Suppl 2:N18-23. 12412773

[cit3] Yu Ning, Donnan Peter T., Murphy Michael J., Leese Graham P. (2008). Epidemiology of primary hyperparathyroidism in Tayside, Scotland, UK. Clinical Endocrinology.

[cit4] Wermers Robert A, Khosla Sundeep, Atkinson Elizabeth J, Achenbach Sara J, Oberg Ann L, Grant Clive S, Melton L Joseph (2006). Incidence of Primary Hyperparathyroidism in Rochester, Minnesota, 1993-2001: An Update on the Changing Epidemiology of the Disease. Journal of Bone and Mineral Research.

[cit5] Mokrysheva Natalya G., Mirnaya Svetlana S., Dobreva Ekaterina A., Maganeva Irina S., Kovaleva Elena V., Krupinova Julia A., Kryukova Irina V., Tevosyan Larisa Kh., Lukyanov Stanislav V., Markina Natalia V., Bondar Irina A., Podprugina Natalia G., Ignatieva Irina A., Shabelnikova Olesia Yu., Dreval Alexander V., Antsiferov Mikhail B., Mel'nichenko Galina A., Dedov Ivan I. (2019). Primary hyperparathyroidism in Russia according to the registry. Problems of Endocrinology.

[cit6] Hunter D. Hyperparathyroidism: Generalized Osteitis Fibrosa with Multiple Osteoclastomata. Proc R Soc Med. 1931;24(4):486-96. 19987984 10.1177/003591573102400461 PMC2182726

[cit7] Aub Joseph C., Albright Fuller, Bauer Walter, Rossmeisl Elsie (2008). STUDIES OF CALCIUM AND PHOSPHORUS METABOLISM. Journal of Clinical Investigation.

[cit8] Bernheim J, Rathaus V, Rathaus M, Bernheim J. Anemia in primary hyperparathyroidism. Nephrologie. 1986;7(1):28-30. [In French] 3960258

[cit9] Boxer M, Ellman L, Geller R, Wang CA. Anemia in primary hyperparathyroidism. Arch Intern Med. 1977;137(5):588-93. 857757

[cit10] Mallette LE, Bilezikian JP, Heath DA, Aurbach GD. Primary hyperparathyroidism: clinical and biochemical features. Medicine (Baltimore). 1974;53(2):127-46. 4361513

[cit11] MALLETTE L. E. (2011). Hyporegenerative Anemia in Primary Hyperparathyroidism. Southern Medical Journal.

[cit12] Falko JM, Guy JT, Smith RE, Mazzaferri EL. Primary hyperparathyroidism and anemia. Arch Intern Med. 1976;136(8):887-9. 949188

[cit13] Prikaz Ministerstva zdravookhraneniya i sotsial'nogo razvitiya Rossiiskoi Federatsii ot 15 fevralya 2006 g. N 87 «Ob utverzhdenii standarta meditsinskoi pomoshchi bol'nym s pervichnym giperparatireozom». Dostupno po: https://docs.cntd.ru/document/901971538. Ssylka aktivna na 25.08.2021.

[cit14] Klinicheskie rekomendatsii Ministerstva zdravookhraneniya Rossiiskoi Federatsii «Pervichnyi giperparatireoz» ot 2020 g. Dostupno po: https://cr.minzdrav.gov.ru/schema/88_4. Ssylka aktivna na 25.08.2021.

[cit15] Haemoglobin concentrations for the diagnosis of anaemia and assessment of severity. Vitamin and Mineral Nutrition Information System. Geneva, World Health Organization, 2011 (WHO/NMH/NHD/MNM/11.1). Available from: http://www.who.int/vmnis/indicators/haemoglobin.pdf. Accessed 25 August 2012.

[cit16] Bhadada Sanjay K., Bhansali Anil, Ahluwalia Jasmina, Chanukya G. V., Behera Arunanshu, Dutta Pinaki (2008). Anaemia and marrow fibrosis in patients with primary hyperparathyroidism before and after curative parathyroidectomy. Clinical Endocrinology.

[cit17] Meytes D, Bogin E, Ma A, Dukes P P, Massry S G (2008). Effect of parathyroid hormone on erythropoiesis.. Journal of Clinical Investigation.

[cit18] Sikole A. (2002). Pathogenesis of anaemia in hyperparathyroidism. Medical Hypotheses.

[cit19] Panjeta Mirsad, Tahirović Ismet, Sofić Emin, Ćorić Jozo, Dervišević Amela (2017). Interpretation of Erythropoietin and Haemoglobin Levels in Patients with Various Stages of Chronic Kidney Disease. Journal of Medical Biochemistry.

[cit20] Kumbasar B., Taylan I., Kazancioglu R., Agan M., Yenigun M., Sar F. (2004). Myelofibrosis Secondary to Hyperparathyroidism. Experimental and Clinical Endocrinology & Diabetes.

[cit21] Gorbacheva A. M., Shklyayev S. S., Eremkina A. K., Bratchikova A. A., Mokrysheva N. G. (2020). Anemia in primary hyperparathyroidism. Russian journal of hematology and transfusiology.

[cit22] Huang Shu-Chuan, Wu Vin-Cent, Chou Guan, Huang Tzu-Yu, Lin Shih-Yi, Sheu Wayne Huey-Herng (2009). Benign Parathyroid Adenoma Presenting with Unusual Parathyroid Crisis, Anemia and Myelofibrosis. Journal of the Formosan Medical Association.

[cit23] Greenfield Edward M., Gornik Sandra A., Horowitz Mark C., Donahue Henry J., Shaw Steven M. (2010). Regulation of cytokine expression in osteoblasts by parathyroid hormone: Rapid stimulation of interleukin-6 and leukemia inhibitory factor mRNA. Journal of Bone and Mineral Research.

[cit24] Liu Jing-Yao, Brass David M., Hoyle Gary W., Brody Arnold R. (2011). TNF-α Receptor Knockout Mice Are Protected from the Fibroproliferative Effects of Inhaled Asbestos Fibers. The American Journal of Pathology.

[cit25] Baradaran A, Nasri H. Intensification of Anemia by Secondary Hyperparathyroidism in Hemodialysis Patients. Med J Islam World Acad Sci. 2001;14(4):161-6.

[cit26] Teng Yirong, Teng Zhaowei, Xu Shuanglan, Zhang Xiguang, Liu Jie, Yue Qiaoning, Zhu Yun, Zeng Yong (2021). The Analysis for Anemia Increasing Fracture Risk. Medical Science Monitor.

[cit27] Chen Zhao, Thomson Cynthia A., Aickin Mikel, Nicholas J. Skye, Van Wyck David, Lewis Cora E., Cauley Jane A., Bassford Tamsen (2010). The Relationship Between Incidence of Fractures and Anemia in Older Multiethnic Women. Journal of the American Geriatrics Society.

[cit28] Valderrábano Rodrigo J., Lee Jennifer, Lui Li-Yung, Hoffman Andrew R., Cummings Steven R., Orwoll Eric S., Wu Joy Y. (2017). Older Men With Anemia Have Increased Fracture Risk Independent of Bone Mineral Density. The Journal of Clinical Endocrinology & Metabolism.

[cit29] Panda Suchismita (2017). Study of Red Cell Fragility in Different Stages of Chronic Kidney Disease in Relation to Parathyroid Hormone. JOURNAL OF CLINICAL AND DIAGNOSTIC RESEARCH.

[cit30] Yilmaz H. (2014). Assessment of mean platelet volume (MPV) in primary hyperparathyroidism: Effects of successful parathyroidectomy on MPV levels. Endocrine Regulations.

